# Comparison of direct and indirect models of early induced acute lung injury

**DOI:** 10.1186/s40635-020-00350-y

**Published:** 2020-12-18

**Authors:** Laura Chimenti, Luis Morales-Quinteros, Ferranda Puig, Marta Camprubi-Rimblas, Raquel Guillamat-Prats, Maria Nieves Gómez, Jessica Tijero, Lluis Blanch, Gustavo Matute-Bello, Antonio Artigas

**Affiliations:** 1 Critical Care Centre, Parc Taulí Hospital Universitari, Institut d’Investigació i Innovació Parc Taulí I3PT, Universitat Autònoma de Barcelona, Parc Taulí 1, 08208 Sabadell, Spain; 2 Hospital Universitari Sagrat Cor., Grupo Quirón Salud, Barcelona, Spain; 3 CIBER de Enfermedades Respiratorias, Instituto de Investigación Carlos III, Madrid, Spain; 4 Medical Research Service of the Veterans Affairs/Puget Sound Health Care System, Seattle, WA USA; 5 Centre for Lung Biology, Division of Pulmonary and Critical Care Medicine, Department of Medicine, University of Washington School of Medicine, Seattle, WA USA

**Keywords:** Acute lung injury, Animal models, Hydrochloric acid, Cecal ligation puncture, Lipopolysaccharide

## Abstract

**Background:**

The animal experimental counterpart of human acute respiratory distress syndrome (ARDS) is acute lung injury (ALI). Most models of ALI involve reproducing the clinical risk factors associated with human ARDS, such as sepsis or acid aspiration; however, none of these models fully replicates human ARDS.

**Aim:**

To compare different experimental animal models of ALI, based on direct or indirect mechanisms of lung injury, to characterize a model which more closely could reproduce the acute phase of human ARDS.

**Materials and methods:**

Adult male Sprague-Dawley rats were subjected to intratracheal instillations of (1) HCl to mimic aspiration of gastric contents; (2) lipopolysaccharide (LPS) to mimic bacterial infection; (3) HCl followed by LPS to mimic aspiration of gastric contents with bacterial superinfection; or (4) cecal ligation and puncture (CLP) to induce peritonitis and mimic sepsis. Rats were sacrificed 24 h after instillations or 24 h after CLP.

**Results:**

At 24 h, rats instilled with LPS or HCl-LPS had increased lung permeability, alveolar neutrophilic recruitment and inflammatory markers (GRO/KC, TNF-α, MCP-1, IL-1β, IL-6). Rats receiving only HCl or subjected to CLP had no evidence of lung injury.

**Conclusions:**

Rat models of ALI induced directly by LPS or HCl-LPS more closely reproduced the acute phase of human ARDS than the CLP model of indirectly induced ALI.

## Introduction

The conceptual model of acute respiratory distress syndrome (ARDS) includes the following: (a) lung inflammation; (b) acute severe hypoxemia; (c) edema due to increased permeability, hyaline membranes, and alveolar hemorrhage. Various pathological processes can cause ARDS either directly through an insult to the parenchyma (pneumonia, aspiration, contusion) or indirectly through an acute systemic inflammatory response (sepsis, trauma, bypass surgery) [1]. The animal experimental counterpart of human ARDS is acute lung injury (ALI). Many animal models of ALI are based on the reproduction of the clinical disorders associated with ARDS in humans; however, none of them fully replicates the human disease [2].

Sepsis from pulmonary or non-pulmonary infections is a major cause of ARDS [3, 4]. In animals, sepsis is induced by systemic administration of lipopolysaccharide (LPS) or live bacteria, or by creating an endogenous infection, for instance, by ligating and puncturing the cecum (cecal ligation and puncture, CLP).

LPS is an important mediator of sepsis in response to gram-negative bacteria, and systemic administration of LPS was one of the earliest approaches to mimic the consequences of bacterial sepsis in animals [2]. Intravenous administration of LPS leads to changes in the lungs, including changes in polymorphonuclear leukocyte (PMN) deformability and PMN entrapment in pulmonary capillaries [5], although only small numbers of PMN migrate into the airway spaces. By contrast, intratracheal administration of LPS results in large increases in PMN in the air spaces [6].

To model ARDS induced by sepsis, CLP is commonly used to produce lung injury secondary to peritonitis [7, 8]. CLP-associated lung injury develops within 18 h to 72 h [9]; it is characterized by hypoxemia, neutrophilic inflammation, and interstitial and alveolar edema [10].

Another common cause of ARDS is the aspiration of gastric contents [11]. Hydrochloric acid (HCl) is used in animal models to reproduce ARDS caused by gastric acid aspiration. This neutrophil-dependent form of lung injury is characterized by damage to the alveolar epithelium, alveolar hemorrhage, and intra-alveolar and interstitial edema [12]. Although under normal conditions, gastric acid prevents bacteria from growing in the stomach, pathogenic organisms may colonize gastric contents when antacids or histamine H2–receptor antagonists increase the pH in the stomach [13, 14] or when patients receive enteral feedings [15, 16]. Thus, both an inflammatory response to the particulate matter and bacterial infection may be responsible for the lung inflammatory response after the aspiration of gastric contents, so a two-hit animal model using HCl and to simulate the gastric contents and LPS to simulate infection might better reflect the risk factors in patients with ARDS.

Our aim was to compare four experimental animal models of ALI to characterize a model which more closely could reproduce the acute phase of human ARDS. Thus, we replicated mechanisms of direct or indirect human ARDS: (1) intratracheal instillation of HCl, to mimic direct ALI due to bronchial aspiration of gastric contents; (2) intratracheal instillation of LPS, to mimic direct ALI due to bacterial infection; (3) intratracheal instillation of HCl and LPS, to mimic direct ALI due to bronchial aspiration of gastric contents with bacterial superinfection; and 4) cecal ligation and puncture (CLP), to induce peritonitis as a single-insult model of indirect ALI.

## Methods

### Animals

We studied 80 pathogen-free male Sprague–Dawley rats (8 weeks old; 250–300 g; Charles River, Chatillon-Sur-Chalaronne, France) housed in light/dark cycle-regulated, air-conditioned (23 °C and 60% relative humidity) quarters with free access to standard food pellets (A04; Panlab, Barcelona, Spain) and tap water. Eight animals/group were used to obtain the BALF and the lung homogenate; 8 animals/group were used for the histological examination and the lung wet/dry weight ratio. The Animal Research Ethics Committee of the Autonomous University of Barcelona (UAB) approved the study.

### Pulmonary models of ALI

Rats were anesthetized with isoflurane (2-5%) and were suspended vertically (90° angle) from their incisors for orotracheal intubation, as described previously [17]. A 0.5-mm-diameter glass tube was guided 1 cm the vocal cords, and 300 μl of one of the following solutions were instilled: (1) HCl (1.5 μl/g, 0.1 N, pH = 1.4) (17); (2) LPS (10 μg/g b.w; *Escherichia coli* O55:B5, Sigma); (3) 0.1 N HCl (150 μl) followed after 30 min by *E. coli* LPS, (10 μg/g b.w.), 150 μl; or (4) saline (0.9% NaCl) solution as a control. Five hundred microliters of air followed the bolus of 300 μl or 150 μl of the solutions, in order to facilitate its arrival and distribution through lungs, in concordance with previous studies regarding intratracheal instillation methods [18]. Rats were sacrificed by exsanguination 24 h after the instillations.

### Extrapulmonary model of ALI

#### Cecal ligation and puncture

Rats were anesthetized by intraperitoneal injection of ketamine (90 mg/kg) and xylazine (10 mg/kg) and given an intraperitoneal fluid bolus of 1 ml 0.9% normal sterile saline as pre-emptive resuscitation. A 2 cm longitudinal incision was made in the lower abdomen; the cecum was exteriorized, and the distal 4 mm was ligated and punctured twice with an 18-gauge needle. The cecum was replaced and the incision closed. Analgesia consisted of buprenorphine (0.1 mg kg^−1^ SC). For the next 24 h, animals were carefully monitored and kept in a warm, well-ventilated environment and were monitored for body temperature, activity, and free access to food and water.

After 24 h of the induction of both the intrapulmonary or the extrapulmonary model of ALI, animals were intraperitoneally anesthetized with an injection of ketamine (90 mg/kg) and xylazine (10 mg/kg) and sacrificed by the exsanguination of the abdominal aorta artery.

### Obtaining and processing bronchoalveolar lavage fluid

After exsanguination, the left main bronchus was tied with a string at the left hilum. Bronchoalveolar lavage fluid (BALF) was obtained from the right lung by connecting a syringe to the cannula placed in the trachea and then flushing through it 5 mL of sterile 0.9% NaCl with 1 mM EDTA five times. BALF recovery was always greater than 85%. Cells in BALF were counted using a hemacytometer (Neubauer, Marienfeld, Lauda-Königshofen, Germany), and slides were prepared by cytocentrifugation (Shandon Cytospin 4, Thermo Electron Corporation, Marietta, OH, USA) and Diff-Quick staining (Pancreac Quimica SAU; Castellar del Vallès, Spain). For each rat, approximately 500 cells were counted. BALF was spun at 800 g for 10 min, and the supernatant was stored at – 80 °C for subsequent analysis.

### Lung wet/dry weight ratio

The left lung was dissected immediately after exsanguination for edema assessment and the wet weight recorded. The lung was then placed in an incubator at 80 °C for 24 h and the dry weight recorded.

### Lung injury scoring

After the animals were sacrificed, lungs were instilled and inflated at 15 cmH_2_O with 4% paraformaldehyde and removed for paraffin embedding. Slices at 4 μm thickness sections were subsequently stained with hematoxylin and eosin (Sigma-Aldrich Ltd.).

Lung injury scores (LIS) were quantified by an investigator blinded to the treatment groups using recently published criteria [19]. As shown in Table 1, the LIS was obtained by the sum of each of the six independent variables: neutrophils in the alveolar space and/or in the interstitial space, presence of hyaline membranes, proteinaceous debris filling the airspaces, alveolar septal thickening, and alveolar congestion. This sum was weighted according to the relevance ascribed to each feature by the American European Consensus Committee [19], and then was normalized to the number of fields evaluated and arbitrarily multiplied by 10 to obtain continuous values between 0 and 10 (both inclusive). Thus, the resulting lung injury score was derived from the following calculation: LIS: {[(20 × A) + (14 × B) + (7 × C) + (7 × D) + (2 × E) + (2 × F)]/(number of fields × 100)} × 10.

**Table 1:** Histologic lung injury results

	Score per field		
Parameter^a^	1	2	3
A. Neutrophils in the alveolar space	None	1–5	> 5
B. Neutrophils in the interstitial space	None	1–5	> 5
C. Hyaline membranes	None	1	> 1
D. Proteinaceous debris filling the airspaces	None	1	> 1
E. Alveolar septal thickening	< 2×	2×–4X	>4×
F. Alveolar congestion	None	1–5	> 5

^a^Lung injury scoring system [adapted from Matute-Bello et al.^18^].

### Cytokine and protein measurements

Total protein concentration in BALF was quantified using the Micro BCA protein assay kit (Pierce, Rockford, IL, USA). Protein was extracted from the lung tissue homogenate with a protease inhibitor cocktail (Roche, Merck Millipore, Darmstadt, Germany) mixed with orthovanadate sodic (1 mM) and lysis buffer solution (25 mM Tris-HCl, pH 7.6, 150 mM NaCl, 1% NP-40, 1% sodium deoxycholate, 0.1% SDS). For cytokine measurements, lung was homogenized in lysis buffer containing 1 mM sodium orthovanadate, protease inhibitor cocktail tablets (1 tablet for 250 mg of lung tissue) (Roche; Mannheim, Germany), 0.5% Triton X-100, 150 mM NaCl, 15 mM Tris, 1 mM CaCl2, and 50 mM MgCl2 (pH 7.4) using a hand-held homogenizer. The homogenates were incubated for 30 min at 4 °C, centrifuged at 12,000 rpm at 4 °C for 20 min, and then filtered with 0.45 μm Nanosep filters (Pall Life Sciences; Madrid, Spain). IL-1β, MCP-1, and VEGF-A in lung homogenate were determined by multiplex assay (Luminex, Affymetrix, Dumbarton Circle Fremont, CA, USA); IL-6, GRO/KC, TNF-α, and IL-10 in lung homogenate were determined by a multiplex assay (Luminex, Merck Millipore, Darmstadt, Germany). PAI-1 in lung homogenate was determined by uniplex assay (Luminex, Merck Millipore, Darmstadt, Germany).

### Statistical analysis

The results of the quantitative variables were expressed as means ± SEM. Comparisons between the experimental rat groups and saline-treated placebo rat group were performed using one-way analysis of variance (ANOVA) followed by the post hoc Dunnett’s tests. When data failed the normality test in the one-way ANOVA, the Kruskal-Wallis one-way ANOVA on ranks was used. A *p* value < 0.05 was considered statistically significant. (StatView 5.0.1; Abacus Concept, Berkeley, CA, USA).

## Results

**Fig. 1:**
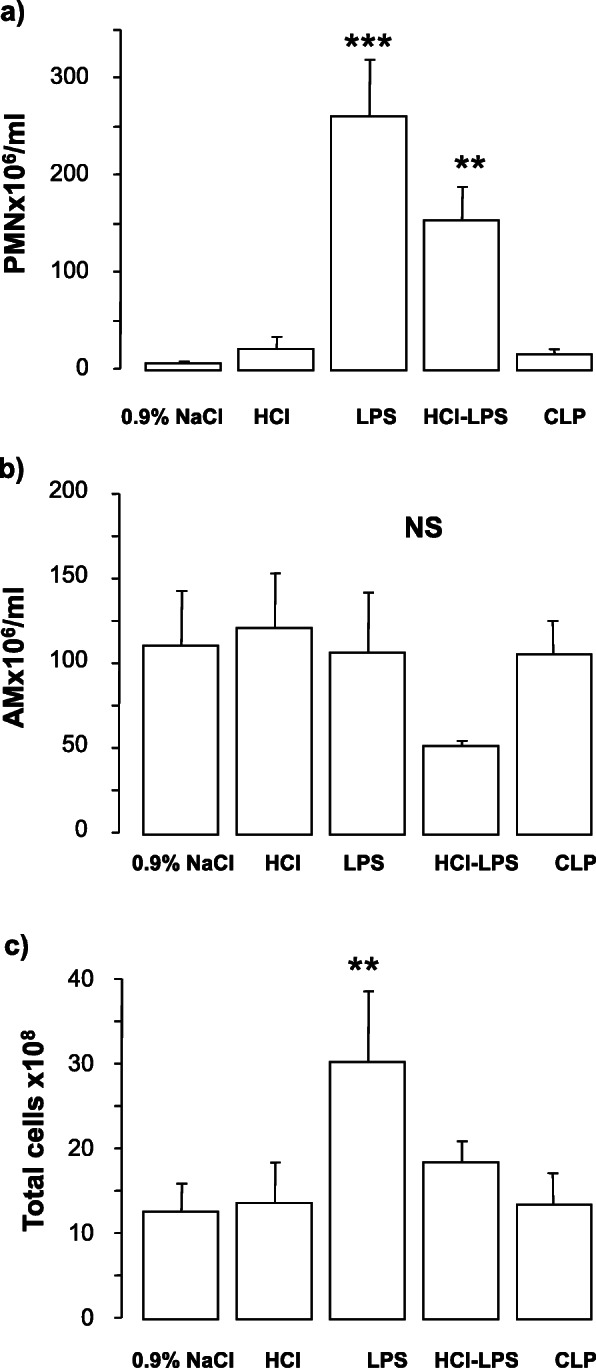
Absolute **a** neutrophil (PMN), **b** macrophage (AM), and **c** total cell counts in the bronchoalveolar lavage fluid of rats 24 h after the induction of the injury. Data are presented as mean±SEM. PMN**,** LPS: ***p < 0.0001 vs 0.9% NaCl 24 h or HCl or CLP; HCl-LPS: **p < 0.005 vs 0.9% NaCl or HCl or CLP; total cells, LPS: **p < 0.001 vs 0.9% NaCl or HCl or CLP

**Fig. 2:**
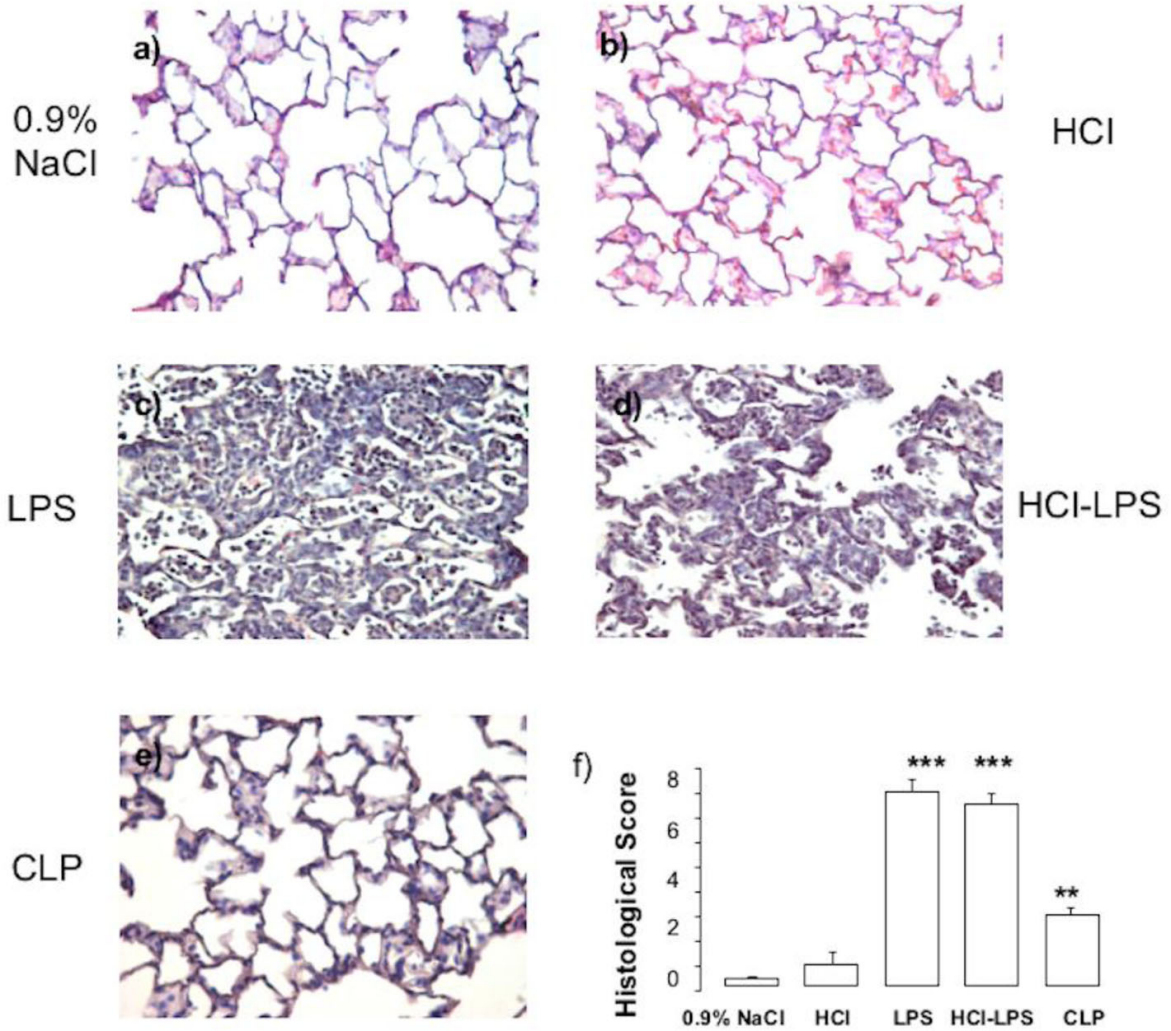
Representative images of **a**–**e** hematoxylin and eosin-stained lung tissue sections. Evidence of lung injury was observed in LPS and HCl-LPS rats (**c** and **d**) while no lung injury was detected in HCl, CLPor 0.9% NaCl rats (**a**, **b**, **e**). **f** Histological score in animals 24 h after induction of the injury. Data are presented as mean ± SEM. LPS or HCl-LPS: ***p < 0.0001 vs 0.9% NaCl, HCl, CLP; CLP: **p < 0.001 vs 0.9% NaCl or HCl

**Fig. 3:**
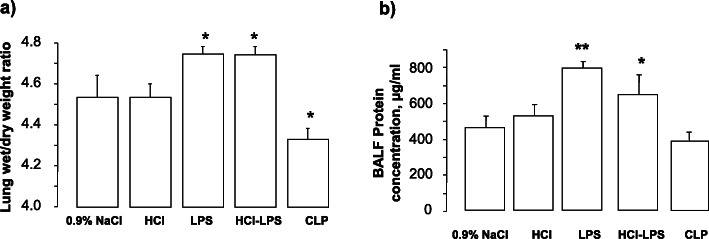
Lung edema, quantified by the **a** wet/dry weight ratio and **b** total proteins from bronchoalveolar lavage fluid. Data are presented as mean ± SEM. Wet/dry weight ratio, LPS: ***p < 0.0001 vs 0.9% NaCl or HCl or CLP; HCl-LPS: **p < 0.005 vs 0.9% NaCl or HCl; total proteins, 24 h, LPS: **p < 0.01 vs 0.9% NaCl or HCl or CLP; HCl-LPS: *p < 0.05 vs 0.9% NaCl or CLP

**Fig. 4:**
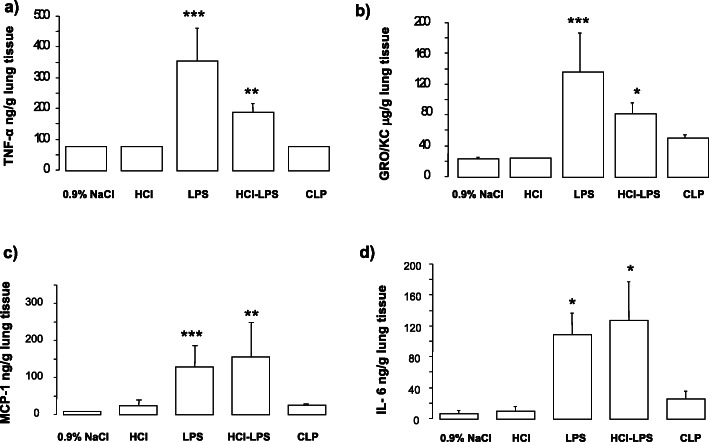
**a** Cytokine TNF-α, **b** GRO/KC, **c** MCP-1, and **d** IL-6 concentrations in lung homogenate of animals 24 h after induction of the injury. Data are presented as mean±SEM. TNF-α, LPS ***p < 0.0001 vs 0.9% NaCl, HCl, or CLP; HCL-LPS: *p < 0.001 vs 0.9% NaCl, HCl, or CLP; GROKC, LPS ***p < 0.0001 vs 0.9% NaCl, HCl, or CLP; HCL-LPS: *p < 0.05 vs 0.9% NaCl, HCl; MCP-1, LPS ***p < 0.001 vs 0.9% NaCl, HCl, or CLP; HCL-LPS: **p < 0.01 vs 0.9% NaCl, HCl, or CLP; IL-6, LPS, and HCL-LPS **p < 0.01 vs 0.9% NaCl, HCl, or CLP

**Fig. 5:**
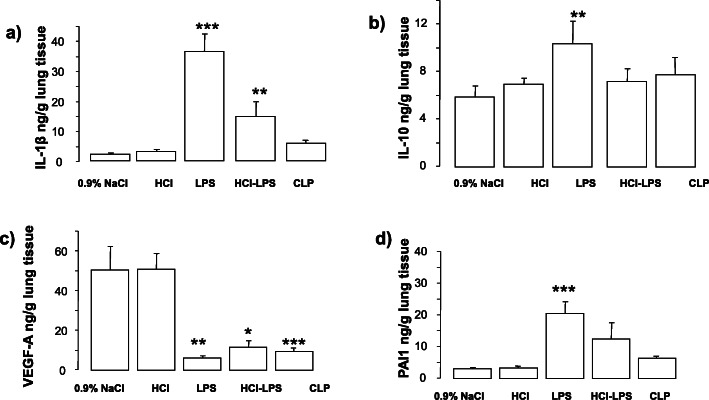
Cytokine IL-1β a, **b** IL-10, **c** VEGFA, and **d** PAI-1 concentrations in lung homogenate of animals 24h after induction of the injury. Data are presented as mean±SEM. IL1 β, LPS ***p < 0.0001 vs 0.9% NaCl, HCl, or CLP; HCL-LPS: **p < 0.01 vs 0.9% NaCl, HCl, or CLP; IL-10, LPS: **p < 0.01 vs 0.9% NaCl; VEGFA, LPS, HCL-LPS, CLP ***p < 0.0001 vs 0.9% NaCl, HCl; PAI-1, LPS: ***p < 0.0001 vs 0.9% NaCl, HCl, or CLP; HCL + LPS: *p < 0.05 vs 0.9% NaCl, HCl, or CLP

Compared to rats in the 0.9% NaCl, HCl, or CLP groups, rats in the LPS and HCL-LPS groups had higher total neutrophil counts in BALF. No neutrophils were found in BALF from CLP rats (Fig. 1a). There were no major differences in total macrophage counts among groups (Fig. 1b). LPS rats showed significantly higher total cell counts than 0.9% NaCl rats (Fig. 1c). Histological analysis of lung tissues detected evidence of lung injury in LPS and HCl-LPS rats. No lung injury was found in HCl, CLP, or 0.9% NaCl rats (Fig. 2). Consistent with the histological findings, the total concentrations of BALF proteins and the wet to dry ratio were significantly greater in LPS and HCl-LPS rats than in HCl, CLP, or 0.9% NaCl rats (Fig. 3a, b, respectively), suggesting changes in the permeability of the alveolo-capillary barrier. Concentrations of the pro-inflammatory cytokines TNF-α and GRO/KC in lung homogenates (Fig. 4a, b, respectively) were greater LPS and HCl-LPS animals compared to 0.9% NaCl rats. Compared to all groups, LPS and HCL-LPS rats had increased MCP-1 and IL-6 (Fig. 4c, d, respectively). LPS and HCl-LPS rats had also higher IL-1β compared to the other groups (Fig. 5a). IL-10 expression in LPS rats was significantly higher than 0.9% NaCl rats (Fig. 5b). VEGF-A concentration was higher in 0.9% NaCl and HCl rats than in LPS, HCl-LPS, or CLP rats (Fig. 5c). PAI-1 levels in LPS and HCl- LPS rats had decreased but remained compared the other groups (Fig. 5d).

## Discussion

Our findings showed that rat models of ALI induced directly by intratracheal instillation of LPS alone or HCl-LPS reproduced more effectively the characteristics of the acute phase of human ARDS than the rat model of ALI induced directly by HCl or indirectly by CLP model at 24 h.

Animal models of ALI have contributed significantly to our understanding of the pathogenesis and pathophysiology of the clinical syndrome of ARDS, however one of the major pitfalls to translation from bench to bedside is that none of the ALI models adequately reproduces the full characteristics of human ARDS [2, 19, 20].

We aimed to compare different experimental animal models of ALI to identify a model which more closely could reproduce human ARDS. To simulate a direct insult to the lung, we instilled HCl, LPS, or HCL followed by LPS into the rat trachea. To simulate an indirect insult, we induced peritonitis by cecal ligation and puncture. Intratracheal instillation of LPS or HCl-LPS was more effective than HCl or CLP in presenting ALI hallmarks at 24 h. Rats instilled with LPS or HCl-LPS had increased W/D ratios compared to the rats in the other groups at 24 h, suggesting more lung edema, likely resulting from increased permeability of the alveolo-capillary barrier. Likewise, histological analysis detected considerable evidence of lung injury in rats administered LPS or HCl-LPS. Moreover, cell and protein analyses of BALF suggested development of ALI in rats administered LPS or HCL-LPS: (1) neutrophils were found to be increased; (2) total protein concentration, a hallmark of leakage in the lung barrier, was also augmented; (3) in lung tissue, concentrations of the cytokines IL-1β, TNF-α, IL-6, GRO/KC, MCP-1, among the most biologically active cytokines in the lungs of patients with ARDS, were increased; (4) PAI-1 levels were increased, suggesting decreased fibrinolytic activity; (5) VEGF-A concentration, a marker of microvascular permeability in ARDS, was decreased; VEGF-A concentration was also decreased in CLP rats. HCl and CLP rats had no lung inflammation or histological changes of lung injury.

Acute respiratory distress syndrome is characterized histopathologically by (a) neutrophilic alveolitis, indicating the presence of an inflammatory response in the alveoli; (b) deposition of hyaline membranes, indicating injury of the alveolar epithelium with disruption of alveolar-capillary barrier, and (c) formation of microthrombi, indicating the presence of endothelial injury [21].

Administration of HCl into the airway is characterized by injury of the airway and alveolar epithelium, including type I alveolar epithelial cells [22, 23] and also injury to the capillary endothelium by a mechanism of involving circulating neutrophils [22, 23]. In a comprehensive mouse model, ALI induced by orotracheal HCL instillation monitored over 10 days, it was observed an early acute neutrophil infiltration matched by a marked increase in BAL neutrophils over 2 days [24]. These findings were reproduced previously in other models of HCL tracheal instillation as early as 4 h [25].

Although our HCl model was based in previous methods [26], we did not observe lung injury at 24 h. In previous studies performed by our group, we observed with a lower concentration of 300 μl of HCl (1.2 μl/g, 0.1 mol/l pH = 1.4) higher protein levels in the bronchoalveolar lavage and raised lung weight at 2 h, but no damage at 24 h [27, 28].

It is known that the concentration and the volume of HCl affect the severity of injury [29]. In the actual study, in order to observe the differences among the administered substance(s), we used the same procedure and volume (0.3 ml) for all the groups, the total volume administered, this was based in our previous experience and literature [30], but also in order to prevent a not desirable injury induced by an excessive volume [31]. Nevertheless, we increased the HCl concentration (1.5 μl/g, 0.1 mol/l pH = 1.4), but we did not found neutrophil infiltration, injury, or increase in inflammatory cytokines in the lungs after intratracheal instillation of HCl alone at 24 h, even though we used a pH and concentration of HCl recommended by recent guidelines [19]. Although the exact mechanism of cellular injury induced by acid instillation is unknown, it is important to highlight that in addition to low pH, gastric contents contain other products that may contribute to the pathogenesis of aspiration-induced lung injury [32]. Although a very low pH is required to produce ALI in animals (pH 1.5), lower than is measured in human gastric contents (pH 3–4), this low pH remains widely used. In addition to this, our results might be explained by a different timing when the experiments were performed rather than the dose used.

LPS is a glycolipid present in the outer membrane of gram-negative bacteria that is composed of a polar lipid head group (lipid A) which responsible for its biologic effects [33]. After LPS exposure, humans and other animals display major features of microvascular lung injury, including leukocyte accumulation in lung tissue, pulmonary edema, profound lung inflammation, and mortality [34]. The intratracheal instillation of LPS is followed by an early phase characterized by increases in PMN, albumin, and pro-inflammatory cytokines in BALF and a later phase 24 h–48 h after instillation characterized by normalization of the BALF cytokine concentrations and increases in the BALF PMN, monocyte, macrophage, and lymphocyte counts [35]. In agreement with these findings, our results showed that in lungs of animals intratracheally administrated with LPS the levels of TNF-α, GRO/KC, MCP-1, and IL-6 early greatly increased. Thus, LPS causes acute pulmonary damage after intranasal or intratracheal administration, but intravenous or intraperitoneal administration does not result in tissue-specific or similar degree of lung injury [36, 37]. LPS is easy to administer and the results tend to be reproducible within experiments. However, LPS instillation does not cause the severe endothelial and epithelial injury that occurs in humans with ARDS [38].

Therefore, LPS by itself provides an incomplete picture of the effects of live bacteria in the lungs, and a two-hit model may better reflect the comorbidities and risk factors present in patients with ALI [39]. Animal models demonstrate that neutrophil recruitment to the lungs increases when hemorrhagic shock is followed by LPS administration, and when sepsis is followed by direct lung injury with immune complexes or LPS [40]. In a two-hit rat model, LPS significantly magnified and prolonged the inflammatory response when administered before acid instillation in the lungs [25]. In another rat model, instillation of acid into the right lung worsened the pathology induced by LPS that was administered 24 h after acid instillation evidenced by worsened oxygenation, increased pulmonary edema, increased TNF-α and cytokine-induced neutrophil chemoattractant production, neutrophil accumulation, and mobilization into the alveolar spaces [41]. In our study, we did not found a synergistic effect between LPS and HCl; the acute inflammatory response and lung injury in rats receiving LPS and HCl in the two-hit model were similar to those observed in rats receiving only LPS. We administered first HCl and after 30 min LPS in order to prevent LPS degradation. Nevertheless, the time interval between the two instillations may not have been long enough to allow HCl to prime the animal and enhance the inflammatory response to LPS, explaining why the injury of HCl and LPS group was not as high as the injury found in the LPS group.

In healthy humans, the protein levels of vascular endothelial growth factor (VEGF) are compartmentalized, with increased levels in the alveolar compartment compared to plasma. The alveolar epithelium is the predominant source of VEGF in the lung, although macrophages and smooth cells also produce VEGF. Compared to healthy controls, patients with ARDS have increased levels of VEGF in plasma but decreased levels in BALF [42], probably due to the breakdown of the alveolar capillary membrane and thus a decrease of VEGF production [43]. We found significantly higher VEGF-A concentration in lung tissues in 0.9% NaCl and HCl rats than in LPS rats, HCl and LPS rats, and CLP rats.

Pulmonary coagulopathy is present in ARDS pathophysiology that may reflect endothelial injury. It is characterized by coagulation activation and reduced fibrinolysis in which different pathways of the coagulation cascade are involved including the regulation of fibrinolysis by the plasminogen activator (PA) and inhibitor pathway (PAI) [44, 45]. High PAI-1 levels have been associated with higher mortality in ARDS [46]. In our study, we found higher levels of PAI-1 in rats instilled with LPS or HCl-LPS compared to control, HCl, and CLP rats, possibly reflecting impairment in the fibrinolytic system.

CLP is the most widely used model of peritonitis. The severity of the injury depends on the number of holes in the cecum and on the size of the needle used to make the holes [47]. Although sepsis is one of the most common causes of ARDS in humans, CLP is usually associated with less impressive intra-alveolar inflammation and hyaline membrane formation [2]. It is probably the single best animal of sepsis rather than that for ALI [48]. In our study, we did not find evidence of lung injury in CLP rats. Lung injury resulting from sepsis models is firstly localized in the vascular and interstitial lung compartments, with little involvement in the alveolar compartment in the first hours. Histological damage looking for hallmarks of ALI demonstrated improved lesion at 24 h in the CLP group [27]. This lesion was not as high as the injury detected in the LPS or the HCL + LPS groups, fact that could be attributed to a longer process for the CLP model as was previously reported by our group [27]. However, we did find hemorrhage and interstitial edema in lung sections and low levels of VEGF-A in lung homogenates of CLP rats. Other studies found decreased VEGF levels in ARDS patients’ lung tissues, and VEGF levels were negatively correlated to apoptotic endothelial cell counts [47]. VEGF is known to promote endothelial survival by inhibiting apoptosis. Together with the histological findings, the low levels of VEGF-A in our study might reflect increased susceptibility to endothelial barrier damage in rats subjected to CLP.

Our study has important limitations. We used only male rats as part of our experiment. Sex differences in preclinical models are becoming increasingly apparent with striking and measurable differences in more than half of the genes’ expression patterns between males and females. In future studies, animal models with females should be performed.

Injurious mechanical ventilation is a major contributor to VALI due to high mechanical stretch. Models using mechanical ventilation with high tidal volumes in conjunction with an indirect hit such as CLP, produce a synergistic effect to lung injury [49]. We did not include mechanical ventilation as part of our models as we aim to have a short model of acute lung injury and we did not want to add hemodynamic instability that would imply adding a third hit.

We did not measure any physiological data such as gas exchange and hemodynamics, as these parameters could be challenging to measure due to the small size of the animal. However, our model fulfilled at least 3 of 4 key features similar to ARDS in humans as recommended by guidelines [19]: (1) histologic evidence of tissue injury, (2) alteration of the alveolar-capillary barrier, and (3) presence of an acute inflammatory response in BALF and serum.

Several limitations are common to models of ALI. Most models are based on one, or, at most, two methods to induce injury, but ARDS in humans is associated with complex interactions between primary risk factors and comorbidities [50]. Experimental ALI models such as acid aspiration and CLP are often used in conjunction with other hits, such as mechanical ventilation, to cause injury and further replicate a clinically relevant picture. In our study, to reproduce human ARDS, we used models mostly based on a single hit. Furthermore, our two-hit HCl-LPS model did not show any synergistic effects between LPS and HCl, probably because the time interval between the two hits was not long enough to allow HCl to prime the animal and increase the inflammatory response to LPS.

## Conclusions

Although no current animal model replicates the complexities of ARDS and, our model does not reproduce the pathologic features of ARDS in humans, namely, diffuse alveolar damage (DAD), our findings demonstrate that rat ALI models induced by a direct insult to the lungs by intratracheal administration of LPS or HCl-LPS are more effective in reproducing the features of the acute phase of human ARDS than rat models of ALI induced by an indirect insult by CLP.

## Data Availability

Data sharing not applicable to this article as no datasets were generated or analyzed during the current study.

## Abbreviations

ALI: Acute lung injury
ARDS: Acute respiratory distress syndrome
BALF: Bronchoalveolar lavage fluid
CLP: Cecal ligation puncture
GRO/KC: Growth KC
HCL: Hydrochloride acid
LPS: Lipopolysaccharide
IL-1β: Interleukin 1 beta
IL-6: Interleukin 6
IL-10: Interleukin 10
LIS: Lung injury score
MCP-1: Monocyte chemoattractant protein-1
NaCl: Sodium chloride
PAI-1: Plasminogen activator inhibitor 1
PMN: Polymorphonuclear
TNF-α: Tumoral necrosis factor alpha
VEGF-A: Vascular endothelial growth factor A
